# Supplementary material for: Impact of semen microbiota on the composition of seminal plasma
Source: Microbiol Spectr. 2024 Feb 13;12(3):e02911-23. doi: 10.1128/spectrum.02911-23 (PMC10913749; doi:10.1128/spectrum.02911-23)
Supplement: Figure S1 — PCA plots of metabolite composition in relation to spermiogram parameters. [file spectrum.02911-23-s0001.docx]

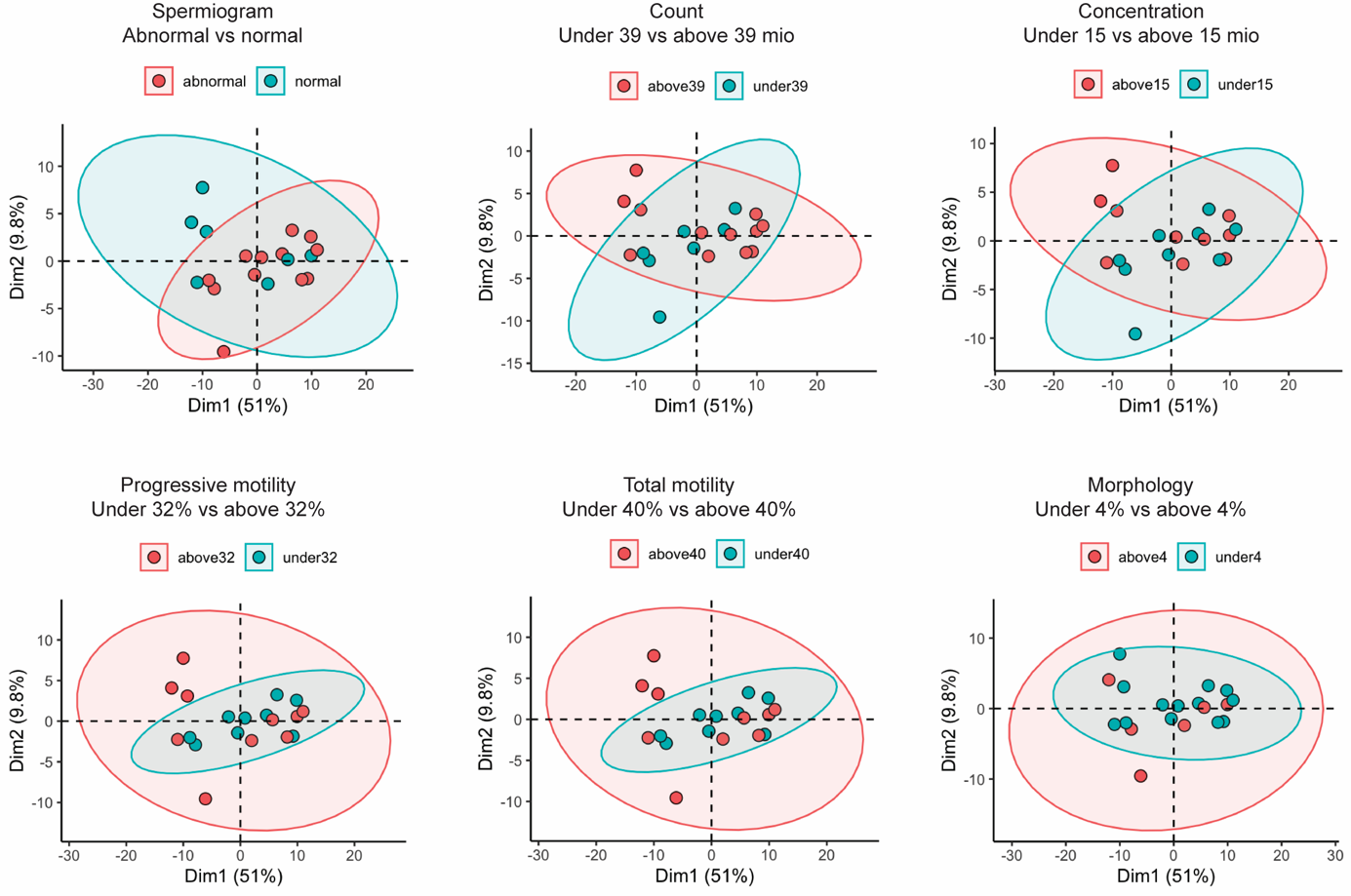


Supplementary figure 1 - Principal Component Analysis (PCA) plots of metabolite composition in relation to spermiogram parameters. The sample points are coloured based on the reference values for spermiogram analysis. The 95% confidence ellipses depict the distribution of samples within each group.
