# Supplementary material for: Impact of semen microbiota on the composition of seminal plasma
Source: Microbiol Spectr. 2024 Feb 13;12(3):e02911-23. doi: 10.1128/spectrum.02911-23 (PMC10913749; doi:10.1128/spectrum.02911-23)
Supplement: Figure S2 — Correlation of relative abundances of bacterial genera and all the metabolites identified in semen. [file spectrum.02911-23-s0002.docx]

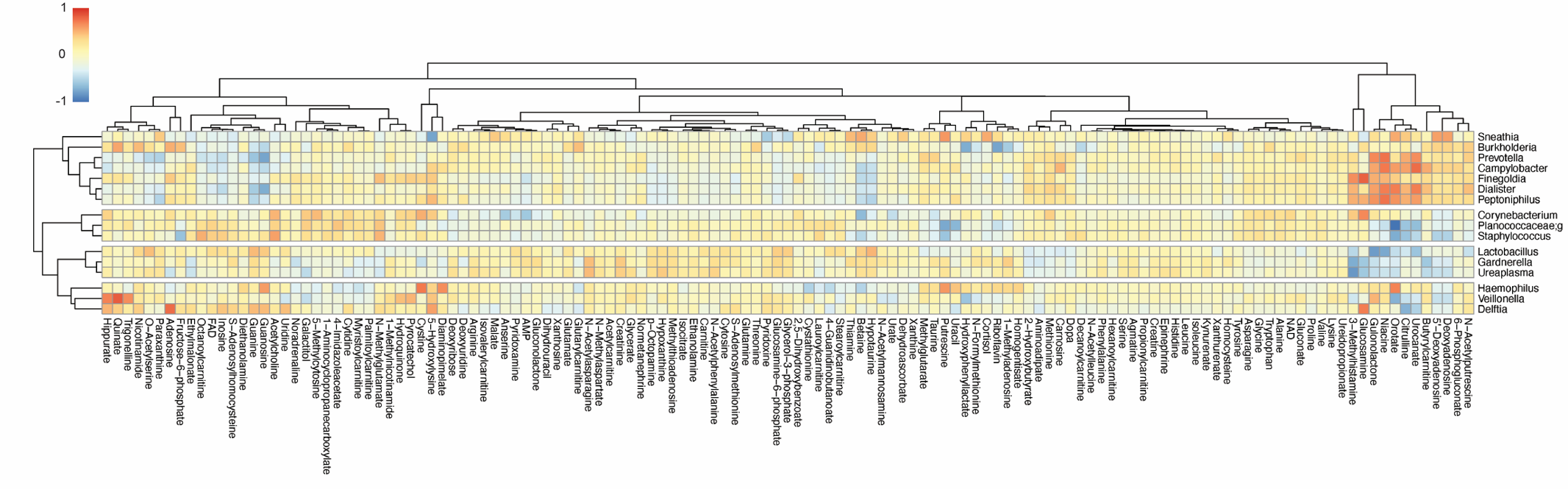


Supplementary figure 2 – Correlation of relative abundancies of bacterial genera and all the metabolites identified in semen. The colour scale indicates positive (red) or negative (blue) correlations.
